# Supplementary material for: Reactive Oxygen Species and Folate Receptor-Targeted Nanophotosensitizers Composed of Folic Acid-Conjugated and Poly(ethylene glycol)-Chlorin e6 Tetramer Having Diselenide Linkages for Targeted Photodynamic Treatment of Cancer Cells
Source: Int J Mol Sci. 2022 Mar 14;23(6):3117. doi: 10.3390/ijms23063117 (PMC8954463; doi:10.3390/ijms23063117)
Supplement: Supplementary file 1 [file ijms-23-03117-s001.zip › ijms-1583709-supplementary.pdf]

Supplementary materials

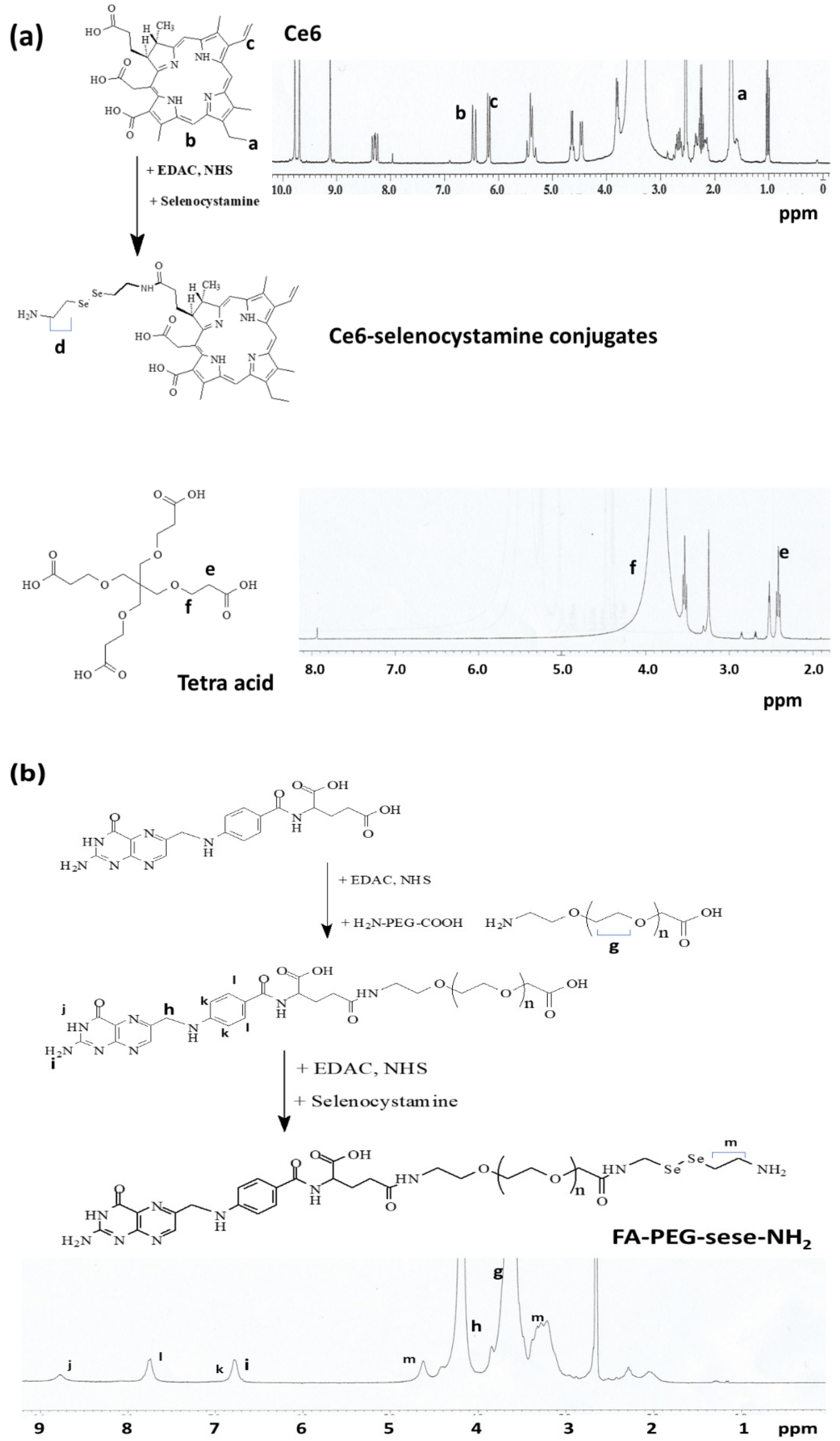

Figure S1. (a) Synthesis scheme and ^{1}H NMR spectra of Ce6, Ce6-selenocystamne (Ce6-sese conjugates) and TA. (b) Synthesis scheme and ^{1}H NMR spectra of FA-PEG-sese-NH_2

Table S1. The effect of H_2O_2 addition on the particle size distribution of FAPEGtaCe6 nanophotosensitizers

H_2O_2 **(mM)**	Particle size distribution (nm)			
	Intensity	Polydispersity index (PDI)	Volume	Polydispersity index (PDI)
0	117.1±45.18	0.244	127.4±47.79	0.228
2	166.8±68.87 (83.6 %)<br>40.98±10.06 (16.4 %)	0.279	169.6±72.56 (84.9 %)<br>37.76±10.67 (15.1 %)	0.280
10	531.5±183.8 (76.9 %)<br>97.91±22.07 (23.1 %)	0.461	425.8±162.7 (87.2)<br>75.67±16.07 (12.8)	0.315

* Particle sizes and PDI indexes were Figure 3 (Intensity %) and Figure S2 (Volume %).

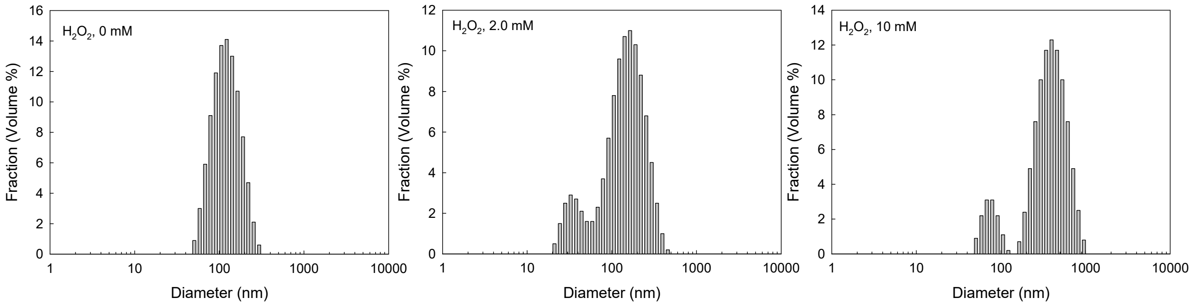

Figure S2. The effect of H_2O_2 concentration on the changes of particle size distribution (volume %). To study ROS sensitivity, nanophotosensitizers in PBS (1 mg/ml) was incubated in the presence of H_2O_2 at 37°C for 3 h.

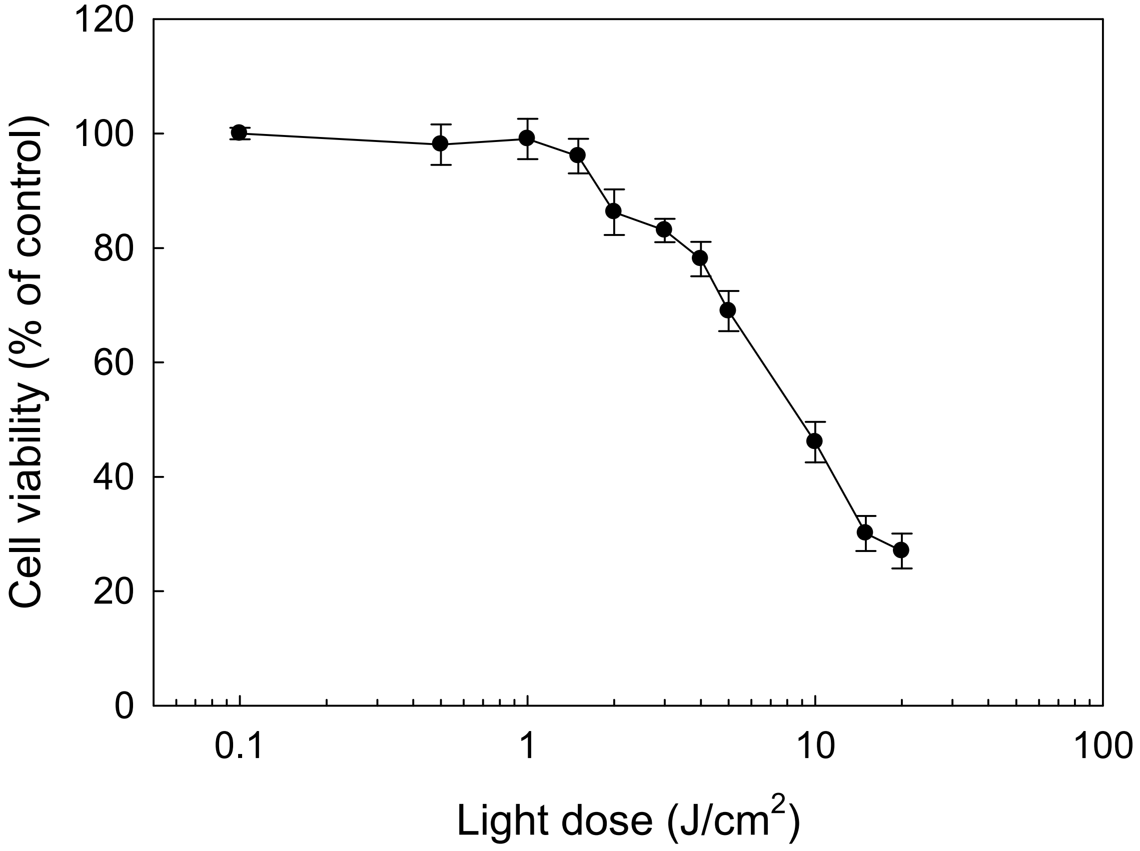

Figure S3. The effect of light dose on the viability of Y79 cells. Ce6 concentration was 2 μg/ml. Y79 cells (2×10^4 cells/well) seeded in 96 well plate were cultured in 5 % CO_2 incubator at 37°C overnight. Following this, Ce6 2 (μg/ml) were treated and then irradiated with various light dose using expanded homogenous beam (SH Systems, Gwangju, Korea). 24 h later, viability of cells was evaluated y MTT assay. Viability of cell at light dose of 0 J/cm^2) was set 100 % as a control and then compared.

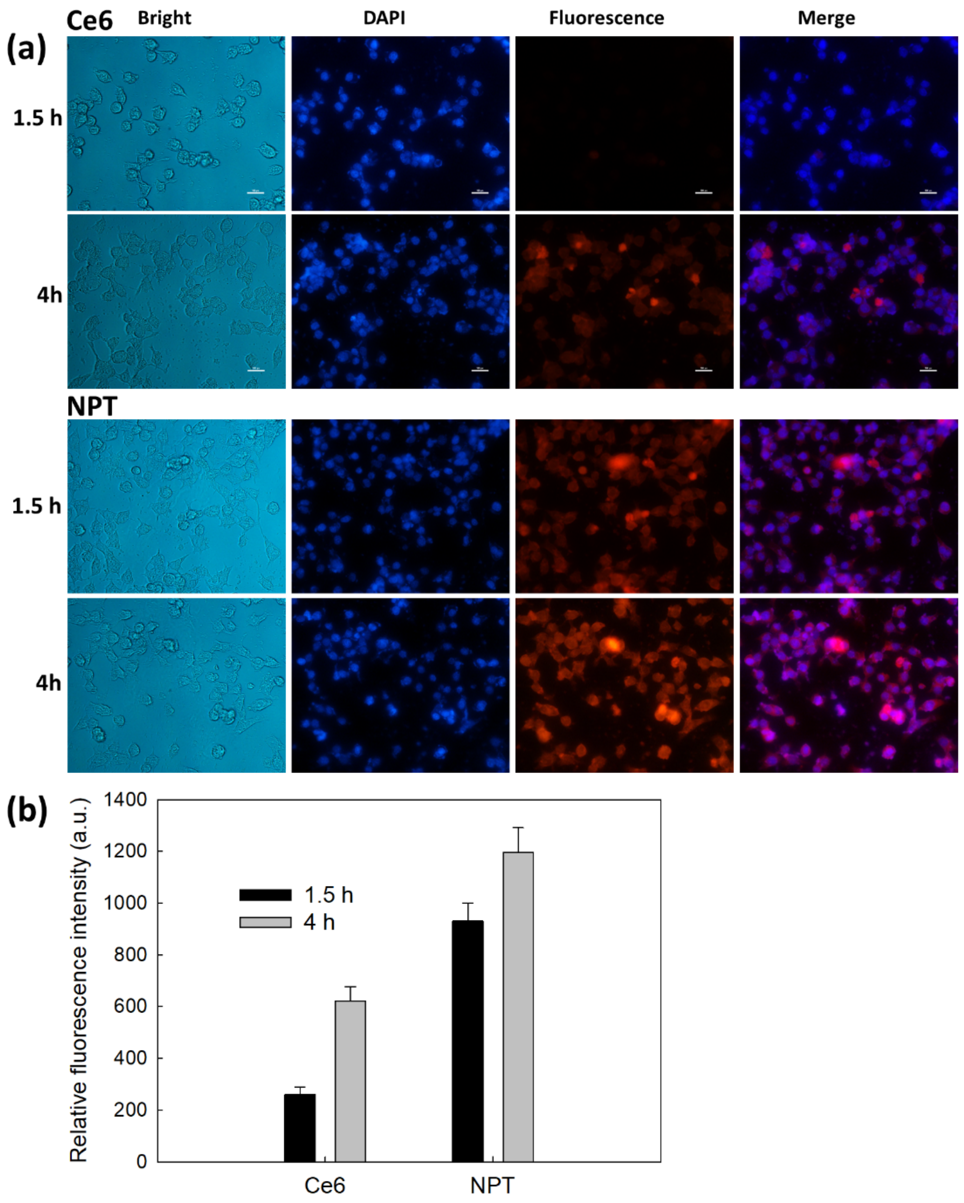

Figure S4. The effect of treatment time of Ce6 and nanophotosensitizers on the intracellular Ce6 uptake of KB cells. (a) Fluorescence observations (b) intracellular Ce6 uptake against various cancer cells (b). Ce6 concentration was 2 μg/mL. Bar = 20 μm.

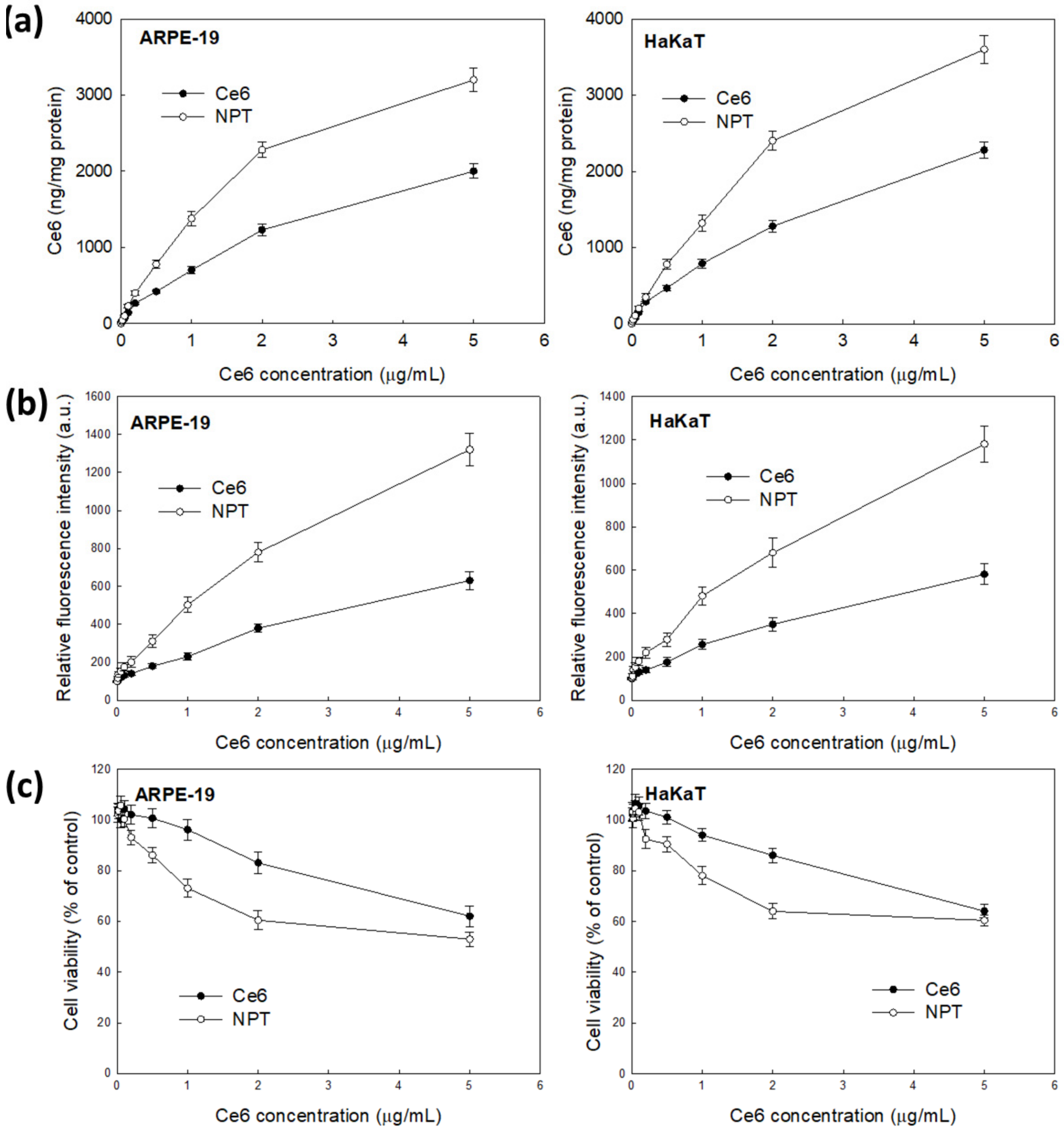

Figure S5. The effect of Ce6 and nanophotosensitizers against non-cancerous normal cell lines. (a) Intracellular Ce6 uptake ratio; (b) intracellular ROS generation; (c) Phototoxicity. All experiments for normal cells were carried out as similar to cancer cells.
